# A Study of Oribatid Mites as Potential Intermediate Hosts of Anoplocephalid Tapeworms of Tatra chamois and Tatra marmots from the Tatra Mountains, Central Europe, and Report of a New Intermediate Host for *Andrya cuniculi*, the Parasite of Leporidae

**DOI:** 10.3390/life13040955

**Published:** 2023-04-06

**Authors:** Alexandra Jászayová, Jana Režnarová, Gabriela Chovancová, Alexei Yu Kostygov, Vyacheslav Yurchenko, Daniela Antolová, Tomasz Zwijacz-Kozica, Alexander Csanády, Zuzana Hurníková

**Affiliations:** 1 Institute of Parasitology, Slovak Academy of Sciences, Hlinkova 3, 040 01 Košice, Slovakia; jaszayova@saske.sk (A.J.); antolova@saske.sk (D.A.); 2 University of Veterinary Medicine and Pharmacy in Košice, Komenského 73, 041 81 Košice, Slovakia; 3 Department of Craniofacial Surgery, Faculty of Medicine, Ostrava University, Syllabova 19, 703 00 Ostrava, Czech Republic; jannakrallova@gmail.com; 4 Administration of Tatra National Park, Tatranská Lomnica 66, 059 60 Vysoké Tatry, Slovakia; gabriela.chovancova@gmail.com; 5 Life Science Research Centre, Faculty of Science, University of Ostrava, Chittussiho 10, 710 00 Ostrava, Czech Republic; kostygov@gmail.com (A.Y.K.); vyacheslav.yurchenko@osu.cz (V.Y.); 6 Zoological Institute of the Russian Academy of Sciences, Universitetskaya 1, 190034 St. Petersburg, Russia; 7 Tatra National Park, Kuźnice 1, 34 500 Zakopane, Poland; tzwijacz@tpn.pl; 8 Department of Biology, Faculty of Humanities and Natural Sciences, University of Prešov, 17. Novembra 1, 080 01 Prešov, Slovakia; alexander.canady@gmail.com

**Keywords:** Anoplocephalidae, *Andrya cuniculi*, chamois, marmot, Oribatida, parasites, Tatra Mountains, *Tectocepheus velatus sarekensis*

## Abstract

Tatra chamois (*Rupicapra rupicapra tatrica* (Blahout 1972)) and Tatra marmot (*Marmota marmota latirostris* (Kratochvíl 1961)) are significant endemic subspecies of the subalpine and alpine ranges of the Tatra Mountains in Central Europe. In four studied localities in the range of their typical biotopes in Slovakia and Poland, we investigated intestinal parasites of Tatra chamois and Tatra marmots, with an emphasis on anoplocephalid tapeworms. We also studied the occurrence, species diversity, and abundance of oribatid mites as intermediate hosts thereof, and the prevalence of cysticercoid larval stages of anoplocephalid tapeworms in collected oribatids using morphological and molecular methods. Coprological analyses revealed the average positivity of *Moniezia* spp. in chamois faeces at 23.5% and *Ctenotaenia marmotae* in marmot samples at 71.1%, with significant differences between the localities under study. Morphological analyses determined the presence of cysticercoids in five oribatid species: *Ceratozetes gracilis*, *Edwardzetes edwardsi*, *Scheloribates laevigatus*, *Trichoribates novus*, and *Tectocepheus velatus sarekensis*. This is the first record of *T. v. sarekensis* as an intermediate host of anoplocephalid tapeworms, as well as the first report of *Andrya cuniculi* occurrence in the territory of the Tatra Mountains, confirmed also by molecular methods.

## 1. Introduction

Tatra chamois (*Rupicapra rupicapra tatrica* Blahout, 1972) and Tatra marmot (*Marmota marmota latirostris* Kratochvíl, 1961) are rare endemic subspecies found in the subalpine and alpine ranges of the Tatra Mountains (Mts.) in Slovakia and Poland, and they are existentially linked only to these habitats. Climatic factors, food availability, and health status, including parasitic infections and predators, have the most serious impact on the abundance and isolation of their populations [1,2]. In terms of health risks, parasitic infections play an important role and can lead to significant depletion of the entire population. So far, the parasitic infections of the Tatra Mts. endemics have been poorly studied in general. Partial research on endoparasites of chamois was carried out in the 1960s and 1980s [1,3,4]; later, the study of Štefančíková et al. [5] focused mainly on lungworms. Recently, Hurníková et al. [6] reported the overall prevalence of gastrointestinal parasites in chamois of the Slovak part of the High Tatra reaching 74.7%. Their most frequent findings were oocysts of *Eimeria* spp. (42.7%), and eggs of *Moniezia* spp. (23.5%); gastrointestinal (GIT) strongylids (7.1%), and *Capillaria* spp. (14%) were detected in lower prevalence. This initial research on gastrointestinal parasites of the Tatra chamois introduced one disputable finding—a significantly higher rate of infection with tapeworms of the family Anoplocephalidae as compared with observations from other European countries. Indeed, these helminths were documented only in 10% of chamois from Croatia [7], in 1% of chamois from the Austrian Alps [8], and rarely in chamois from the Catalan Pyrenees [9], while no positive cases were reported from the Italian Alps [10]. Since the life cycle of anoplocephalids includes oribatid mites, which serve as intermediate hosts, several biotic and environmental factors might contribute to the differences in the prevalence. For example, a windstorm and an extensive fire that occurred in all areas of the Tatra Mts. in 2004 and 2005 led to a significant increase in the species diversity of mites and a higher abundance of moss mites (Oribatida) [11].

Here, we estimated the occurrence, species diversity, and abundance of oribatids in habitats typical for chamois and marmots in the alpine vegetation zone of the Tatra Mountains as well as the prevalence of cysticercoid larval stages of anoplocephalid tapeworms in the mites using molecular methods. In addition, we performed in that region a screening of intestinal parasites in the feces of chamois and marmots.

## 2. Materials and Methods

### 2.1. Study Localities

The Tatra Mountains form the highest part of the Carpathian Mountains. They are located in the northern part of Slovakia on the border with Poland and represent the only mountain range of alpine type in these countries. The mountains cover an area of 786 km^2^, of which 610 km^2^ are in Slovakia, and 176 km^2^ in Poland. The geomorphological unit is divided into two subunits: the Western Tatra and the Eastern Tatra, which are subdivided into the High Tatra and the Belianske Tatra.

**Figure 1:**
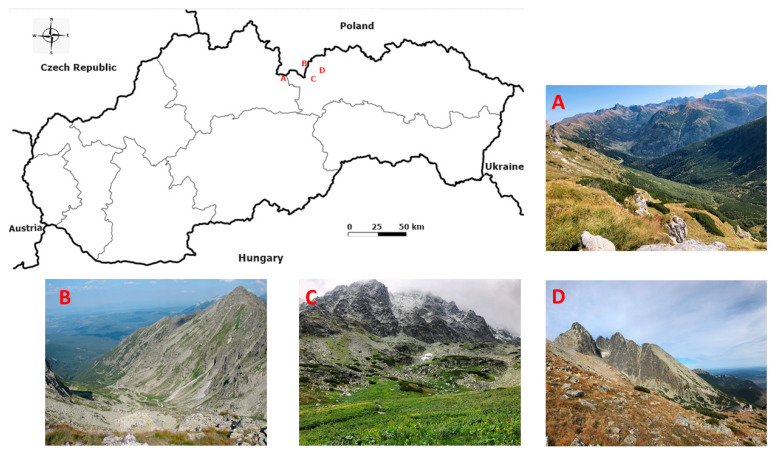
Location of four study sites in the Tatra Mountains with details of their soil profiles where soil samples were sampled; (**A**)—Tomanovská dolina (Photo: A. Jászayová), (**B**)—Dolina Waksmundzka (Photo: T. Zwijacz-Kozica), (**C**)—Velická dolina (Photo: A. Jászayová), (**D**)—Lomnické sedlo (Photo: A. Jászayová).

The localities under study are situated in the alpine zone of the Tatra Mts.: Lomnické sedlo, Velická dolina, Tomanovská dolina (Slovak part of Tatra Mts.), and Dolina Waksmundzka (Polish part of Tatra Mts.) (Figure 1, Table 1).

Lomnické sedlo (LS) (49°11′369″ N, 020°12′974″ E) is a wide, partly grassy saddle between the southeast ridge of the Lomnický štít peak and the Lomnický hrebeň ridge above the valleys Malá Studená dolina and Skalnatá dolina in the High Tatra [12]. The soil profile consists of granitic bedrock with an admixture of grassy areas and stony parts. Permanent subalpine and alpine vegetation, dominated by stands of mountain pine–slash pine (Pinus mugo), extends up to 1800 m asl. Soil samples were collected from the eastern part of the slope, which serves for skiing in winter. Chamois herds occur at the site.

Velická dolina (VD) (49°09′959″ N, 020°09′041″ E) is a terraced valley closed by the lateral spur of the peak of Gerlachovský štít, the section of the main ridge of the High Tatra between the Západný Gerlachovský štít and the Východná Vysoká and the main axis of the spur of the peak Slavkovský štít up to the Bradavica and its shoulder Velické granáty [12]. The subsoil of the site is granitic. In the zone of spruce forests (1300 m asl), the monotonous grassland tall herb community is represented. Soil samples were collected from the grassy valley part and rock formations—marmot toilets. The marmot burrows are located in the Kvetnica section. Chamois herds also occur in the locality.

Tomanovská dolina (TD) (49°13′420″ N, 019°54′299″ E) is situated in the Slovak part of the Western Tatra Mts. The soil profile consists of limestone and dolomitic sediments and the southern parts are made up of crystalline rocks. The limestone bedrock is dominated by contact vegetation of alpine meadows and calciphilous vegetation. There are marmot burrows in the locality, but in the period under study, we did not find any fresh residential tracks or faeces. Chamois herds occur on the site.

Dolina Waksmundzka (DW) (49°23′291″ N, 20°04′836″ E) is situated in the Polish part of the Tatra Mts. The valley is drained by the Waksmundzki Potok brook, a tributary of the Białka river [12]. The soil profile consists of granitic bedrock with an admixture of granitoid with potassium feldspar crystals. High mountain acidophilous grasslands occur here. Samples were taken from sites on granitic bedrock composed of pegmatite and aplite, as well as from sites with granodiorite of equigranular grey tonality. Chamois herds occur at the site.

### 2.2. Faeces Collection and Coprological Analyses

Collections of faecal and soil material were authorized by the Ministry of Environment of the Slovak Republic under permit No. 498/2018-6.3, the Ministry of Climate of Poland (Dz.Urz.MK.2020.6), and the Ministry of Climate and Environment of Poland (Dz.Urz.MKiŚ.2020.25). Chamois and marmot faecal samples from the four studied localities were taken monthly from June to October 2020 and 2021. Due to the COVID-19 pandemic and district-to-district movement bans, it was impossible to collect material at regular monthly intervals in the localities TD and DW, where the number of samplings was reduced. The collected faecal material was stored at +4 °C and examined within 48 h. Since the field samples varied in condition and quality, the conventional qualitative centrifugation–flotation method and the standard sedimentation technique were used for the detection of oocysts and eggs. The identification of these propagative stages was performed based on morphology.

### 2.3. Mite Sampling and Morphological Identification

Soil mesofauna was obtained by collecting soil samples at the studied localities from plots of 10 by 10 m at monthly intervals at random from the sites, where chamois or marmot faeces were found. Ten soil samples weighing about 350 g each were taken from a depth of up to 5 cm depending on the soil profile. Humidity and temperature were measured using a Testo 635-1 thermo-hygrometer (Testo SE & Co. KGaA, Titisee-Neustadt, Germany), and for each site, 10 values measured during each month per the collection period (May to October) were averaged. Due to the COVID-19 pandemic and district-to-district movement bans, it was impossible to collect material at regular monthly intervals in the localities TD and DW, where the number of samplings was reduced.

The collected organic material was dried in a Berlese–Tullgren apparatus for 4 days in the laboratory at the Research Station of the TANAP State Forests in Tatranská Lomnica. Individual organisms were separated using a stereomicroscope Olympus SZX7 at magnifications of 6–140×. The collected mites were cleared with 60% lactic acid for 3–4 days and subsequently identified under the Olympus BX-53 microscope following the determination keys by [13] and nomenclature from [14]. Subsequently, the material was preserved in 80% ethyl alcohol. The abundance of oribatid mites was calculated as the Oribatida individuals (ex.) per 1 m^2^ found at a particular study site during the two years.

### 2.4. Molecular Identification and Phylogenetic Analysis

The gDNA isolation from pools containing 10 morphologically similar individuals followed a previously described non-destructive protocol [15] using the DNeasyBlood & Tissue Kit (Qiagen^®^, Hilden, Germany). For the detection and identification of anoplocephalids, the newly designed specific primers 2256F (5′-tacaatggcggtgtcaacga-3′) and 323R (5′-ttggtcgtcttctcagcacc-3′) were used to amplify a 277 bp-long fragment of the 18S rRNA gene. In pooled samples that tested positive, a fragment of oribatid 18S rRNA gene (~380 bp) was amplified using the primers MbRF (5′-cggagagggagcctgagaaa-3′)—MeR (5′-tgagcactctaattttttcaagtaacg-3′). Thermocycler parameters were set to 94 °C for 5 min, 94 °C for 1 min, 55 °C for 30 s (followed by 35 cycles), 72 °C for 30 s, and 72 °C for 5 min. The obtained PCR products were subsequently sequenced at Eurofins Scientific (Luxembourg City, Luxembourg) using the amplification primers. The obtained sequences were submitted to GenBank under accession numbers: OQ625432 (cestode 178), OQ625433 (cestode 189), OQ625434 (mite 178), and OQ62543 (mite 189).

The nucleotide sequences of cestodes obtained in this work, along with fifteen related ones retrieved from GenBank, were aligned by MAFFT v. 7.490 using the E-INS-I algorithm [16]. A maximum likelihood tree was inferred in IQ-TREE v. 2.2.0 [17] under the K2P + I substitution model, as automatically selected by the built-in ModelFinder module [18]. Branch support was assessed using the ultrafast bootstrap method. The phylogenetic tree was visualized in MEGA 11 [19].

### 2.5. Statistical Analyses

The statistical analyses were performed by the Quantitative Parasitology on the Web [20]. The positivity values were calculated along with 95% confidence intervals (95% CI). The chi-square test was used to test the differences between the total prevalence of parasite propagative stages, the positivity of samples from different localities, and the prevalence of individual parasite taxa in 2020 and 2021. The *p*-values below 0.05 were considered significant. Detailed statistical analyses were performed only for the tapeworms of the family Anoplocephalidae.

The analysis of environmental factors (soil temperature and humidity) for two study sites, where sufficient data have been collected (LS, VD), was conducted in the R environment (v 4.1.3) [21], and for data manipulation and visualization, the “tidyverse” package [22] was used.

The similarity between soil samples in terms of mite species diversity and abundance was analyzed on two study sites (LS, VD) using nonmetric multidimensional scaling (nMDS) with the metaMDS function from the “vegan” package [23]. As input data for nMDS analysis, a dissimilarity matrix was constructed using the Bray–Curtis index. The influence of various factors, including year, month, and location, on sample clustering on the nMDS plot was tested using the Mantel test, which was performed using the mantel function. As input for the Mantel test, the same Bray–Curtis matrix was utilised. Additionally, the daisy function from the “cluster” package [24] was used to construct a Gower similarity matrix for each factor. Finally, the adonis2 function was used to perform permutational multivariate analysis of variance (PERMANOVA) to test for differences in mite associations between study locations.

The study employed generalized linear mixed models (GLMMs) to investigate the impact of location on temperature and humidity. Before analysis, the normality of the distribution of temperature and humidity variables was checked using the Shapiro–Wilk test. Additionally, the check distribution function from the ‘performance’ package [25] was used to evaluate the distribution of variables. Two GLMMs were then fitted using the glmmTMB function from the ‘glmmTMB’ package [26]. Tweedie distribution was selected as the distributional assumption for the response variable. Temperature or humidity was used as the dependent variable, the location was included as a fixed effect, and year and month were included as random effects. Finally, to assess the effect size of the location predictor, the effect function from the ‘effects’ package [27] was applied.

## 3. Results

### 3.1. Coprological Analyses

During the study period in 2020 and 2021, a total of 302 chamois and 39 marmot faecal samples were collected and examined for the presence of intestinal parasites’ propagative stages. Coprological examinations revealed the presence of such stages in 44.37% of examined chamois and in 82.1% of marmot faecal samples. In chamois, the most frequent were oocysts of *Eimeria* spp., which were present in 44.03% of samples, followed by eggs of *Moniezia* spp. (23.5%) and eggs of family Trichostrongylidae (20.2%) (Table 1). In marmot faeces, *Ctenotaenia marmotae* eggs were the most prevalent (71.1%), followed by *Eimeria* spp. oocysts (60.5%) and Trichostrongylidae eggs (7.9%). During the period under study, no statistically significant fluctuation in parasite occurrence was observed concerning season and year.

When comparing the occurrence of propagative stages of GIT parasites in chamois in 2020 and 2021, we found statistically significant differences (*p* = 0.0003). Further statistical analyses of *Moniezia* spp. between localities and years of the study showed significant differences. The positivity was significantly higher in 2020 compared with 2021 (*p* < 0.05). Regarding localities, the occurrence of *Moniezia* spp. eggs was significantly higher in the LS study site than in VD and TD (*p* = 0.002 and *p* = 0.0006, respectively), whereas no difference was found between LS and DW. The tapeworm eggs were also significantly more frequent in the DW study site than in TD and VD (*p* = 0.0013 and 0.0002, respectively). No significant statistical difference was found between TD and VD sites.

In marmot samples, differences in positivity for GIT parasites (*Eimeria* spp., Trichostrongylidae, and *Ctenotaenia marmotae*) propagative stages over the whole study period were statistically highly significant (*p* < 0.0001). The sampling was carried out at only one study site with marmot occurrence (VD); therefore, it is not possible to compare individual study sites with each other. Differences in the occurrence of *Ctenotaenia* spp. between 2020 and 2021 were not significant (*p* = 0.51).

**Figure 2:**
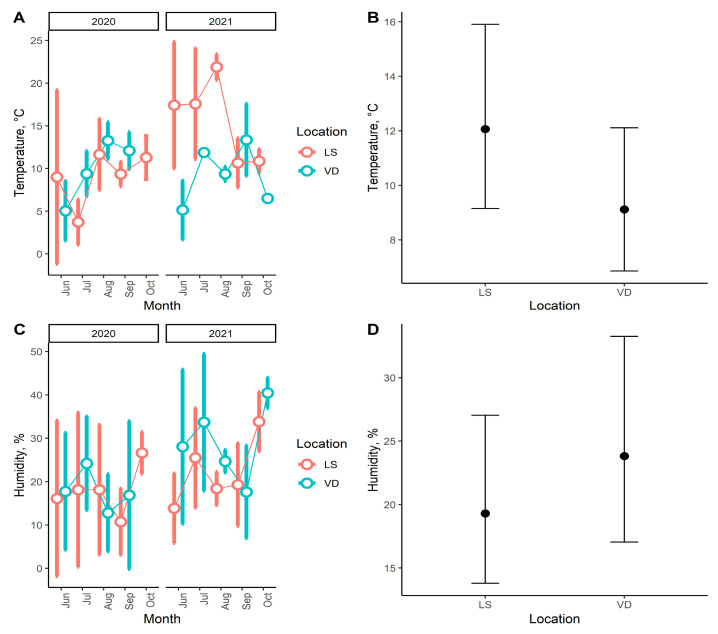
The figures illustrate the descriptive characteristics of two climate variables, temperature (**A**) and soil humidity (**C**). Each dot on the figures represents the values of these variables for each month of observation, while the error bars indicate the standard deviation range. The colour scheme used in the figures (**A**,**C**) differentiates the locations from which the data were collected. The figures (**B**,**D**) show the effect size of the location factor on temperature and humidity, respectively. The dots represent an estimated average effect of the location factor on the dependent variable of GLMMs, while the error bars indicate the 95% confidence intervals of the parameters.

The statistical analysis of the influence of temperature and humidity factors in individual study sites was only possible from two localities (LS and VD), where the sampling was carried out at monthly intervals. Figure 2A,C offers insights into soil temperature and humidity fluctuation across various locations and over time through descriptive statistics. Overall, the visualization reveals that temperature variation tends to be more pronounced than humidity fluctuation, though no discernible pattern emerges. Furthermore, the Shapiro–Wilk normality test results indicate that neither temperature nor humidity variables follow a normal distribution. This is supported by the low *p*-values obtained during the Shapiro–Wilk tests (*p* < 0.05). Additional analysis reveals that the Tweedie distribution best fits both variables. Based on the GLMMs results, it can be concluded that the locality factor has a significant impact on both temperature (GLMM: β_VD = −0.3, z = 3.6, *p* < 0.016) and humidity (GLMM: β_VD = 0.2, z = 2.4, *p* = 0.016). However, it is important to note that the estimation of the effects size of the factor for both models shows that the effects size confidence intervals of the factor overlapped (Figure 2B,D). This suggests that there are no systematic differences between the two localities in terms of soil temperature and humidity.

### 3.2. Morphological Analyses of Mites

**Figure 3:**
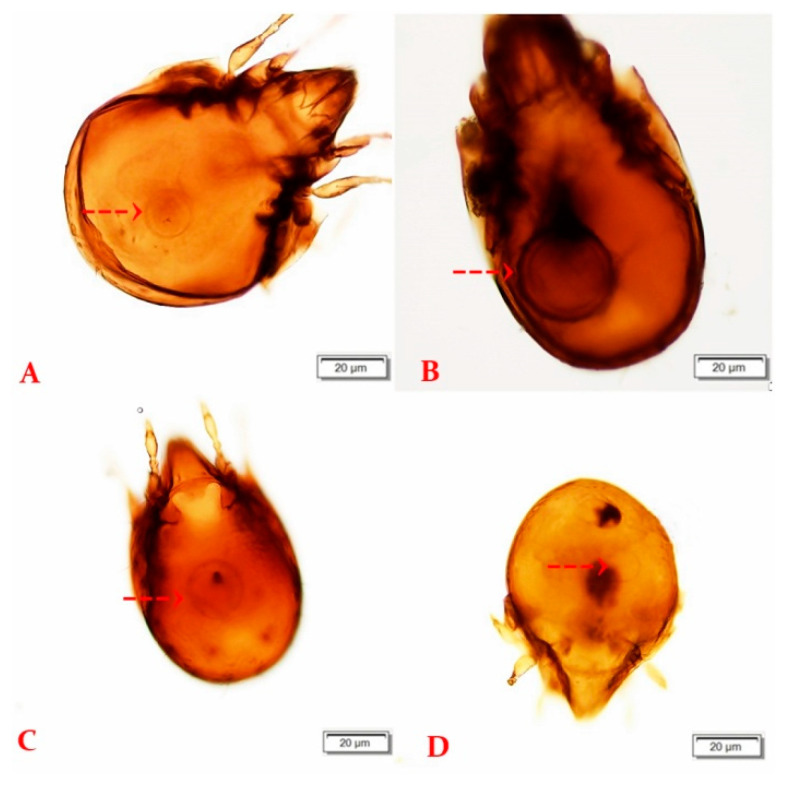
Oribatids with cysticercoid in body cavity ((**A**) *Edwardzetes edwardsi*, (**B**) *Trichoribates novus*, (**C**) *Ceratozetes gracilis*, (**D**) *Scheloribates laevigatus*). The arrow indicates cysticercoid (scale 20 µm).

During the two-year study period, we collected a total of 19,236 oribatid mite specimens. All individuals were morphologically identified and examined for the presence of the larval stage of tapeworms. In total, 99 species and 33 families of Oribatida were recorded at four study sites in 2020 and 2021. The most abundant families were Tectocepheidae (4041 specimens, 3 species), Scheloribatidae (2140 specimens, 7 species), Oppiidae (1930 specimens, 13 species), Ceratozetidae (1744 specimens, 14 species), and Hermanniidae (1278 specimens, 1 species). The presence of 16 species, previously described as intermediate hosts of tapeworms, was confirmed during our study, as seen in Table 2. Morphological analyses determined the presence of cysticercoids in five species*: Ceratozetes gracilis, Edwardzetes edwardsi, Scheloribates laevigatus, Trichoribates novus*, and *Tectocepheus velatus sarekensis* (Figure 3). The latter species has not yet been described as an intermediate host of anoplocephalid tapeworms. Infected individuals were collected at the sites TD (*Tectocepheus velatus sarekensis*—2 ex., *Trichoribates novus*—1 ex.), VD (*Edwardzetes edwardsi*—1 ex., *Scheloribates laevigatus*—1 ex.), and DW (*Ceratozetes gracilis*—1 ex.).

**Figure 4:**
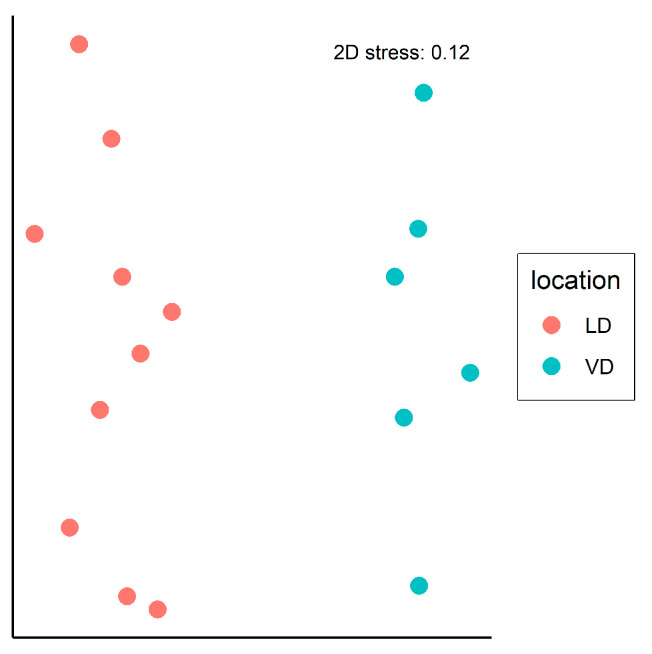
The nonmetric multidimensional scaling (nMDS) plot shows similarities in mites species composition and abundance between soil samples collected from two sites. Each dot represents a soil sample, and the colour indicates the sampling location. The closer the dots are to each other, the more similar their quality and quantity characteristics are.

Regarding the species diversity in individual studied localities, the greatest species richness was recorded at the sites VD (74 species) and LS (72 species), followed by TD (68 species) and DW (50 species). The oribatid mite abundance was the highest at the LS locality (7011 ex.), slightly lower in the VD (6497 ex.), and markedly lower in the sites TD and DW (2930 ex. and 2799 ex., respectively) (Table 1). Because of the movement restrictions due to the COVID-19 pandemic, it was not possible to collect material at regular monthly intervals at the last two sites, and the number of samplings was reduced; therefore, the statistical analyses were performed only for two sites—LS and VD. The comparison of the oribatid mites’ associations between these two localities using the nMDS analysis revealed that the samples clustered into two distinct groups (Figure 2). Further research using Mantel tests showed no significant correlation between the dissimilarity matrix and the potential explanatory variables, namely, months (Mantel: r = −0.025, *p* = 0.594) or years (Mantel: r = −0.014, *p* = 0.492). However, there was a significant correlation between the dissimilarity matrix and the location variable (Mantel: r = 0.621, *p* = 0.002). This indicates that the location factor may be necessary for explaining the observed clustering pattern in the nMDS plot (Figure 4). The results of the PERMANOVA test also indicate that the effect of the location variable (PERMANOVA: R^2^= 0.26, F = 4.972, *p* = 0.001) on the composition of oribatid mites association is unlikely to have occurred by chance alone. The test also explains an essential proportion (26%) of the variation in the data. Overall, the results suggest that the location variable is an influential factor in explaining the peculiarities of the oribatid mites association in this study.

### 3.3. Molecular Analyses

**Figure 5:**
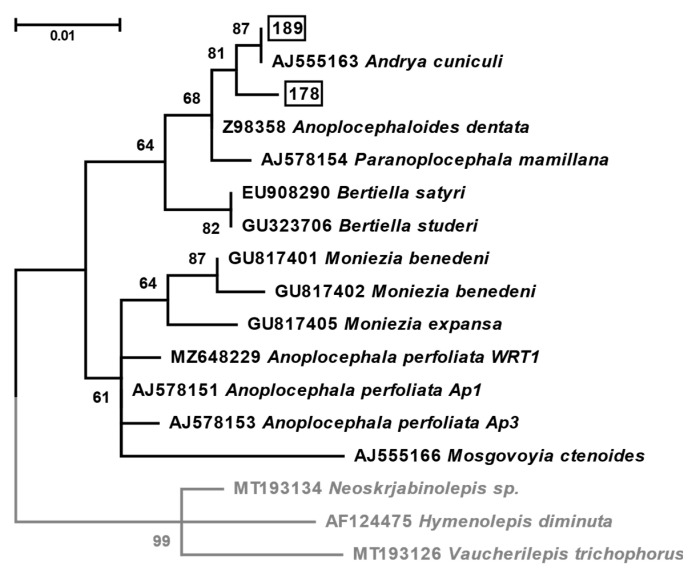
Maximum likelihood phylogenetic tree of Anoplocephalidae based on 18S rRNA gene sequences. The two sequences obtained in this study are boxed. The tree is rooted using Hymenolepididae as an outgroup (shown in grey). The numbers at branches show ultrafast bootstrap supports (values below 50 are omitted). The scale bar denotes the number of substitutions per site.

**Figure 6:**
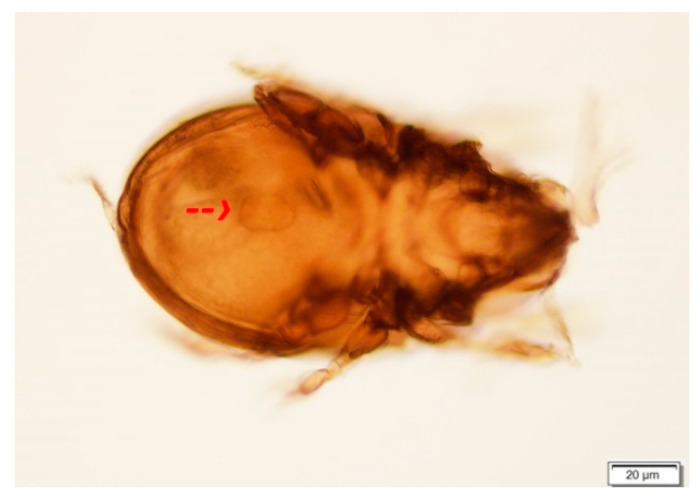
*Tectocepheus velatus sarekensis* with cysticercoid in the body of cavity after DNA isolation and morphological determination. The arrow indicates cysticercoid.

Out of 630 pooled mite samples (5466 specimens) from the four study sites in the Tatra Mts. analysed by PCR with primers specific for DNA of anoplocephalids, two samples (0.0374%) confirmed the presence of these helminths. The analysis of these sequences by BLAST confirmed their affiliation with Anoplocephalidae. Moreover, the sequence of the sample 189 was completely identical to that of *Andrya cuniculi* (AJ555163), while the second had 2 substitutions 0.7% difference. The phylogenetic analysis based on these sequences confirmed their close relatedness to *Andrya cuniculi* (Figure 5). The mites in both positive samples were unambiguously identified by morphological and molecular data (100% identity to KX397637, GQ864284, EF093777, and EF093776) as *Tectocepheus velatus sarekensis* (Figure 6).

## 4. Discussion

Oribatid mites are one of the most abundant arthropod groups in soils with about 10,000 described species. Since 1937, when oribatids were first confirmed as intermediate hosts of sheep tapeworms [32], more than 127 species of them have been identified in the life cycle of more than 27 species of anoplocephalid cestodes [28,29,33]. Oribatid mites are saprophagous; they act as decomposers of dead organic matter and represent suitable intermediate hosts for trophically transmitted parasites since they live in stable and abundant populations in different types of habitats. They ingest tapeworm eggs at random during feeding on the faeces of a definitive host and become infected after they mechanically destroy the outer shell of the egg. The oncosphere released from the egg subsequently develops as a larva–cysticercoid in the oribatid body cavity. An herbivorous definitive host becomes infected following accidental ingestion of infected mites while grazing [31].

The prevalence of infected mites in natural conditions is in general very low. In our study, we microscopically detected six infected specimens (0.03%) of oribatid mites belonging to five species, namely, *Ceratozetes gracilis*, *Edwardzetes edwardsi*, *Scheloribates laevigatus*, *Trichoribates novus*, and *Tectocepheus velatus sarekensis*. To our knowledge, the latest species has not yet been described as an intermediate host of Anoplocephalidae. The main morphological factors enabling oribatid mites to act as intermediate hosts are the body size, the size of the mouth opening, and the structure of the mouth parts. Kassai and Mahunka [34] documented that oribatid species of a body size 300–500 µm could be suitable intermediate hosts. A recent study by Akrami et al. [35] reported that larger species contain more cysticercoids compared to the smaller ones, but smaller mites could be also infected. *Tectocepheus velatus sarekensis* with a body length of 240–320 µm is rather at the lower size limit, but we found two infected individuals suggesting that it is a suitable intermediate host, as was also confirmed via molecular methods. Schuster at al. [36] found that, in larger mites, the oncospheres do not develop as well as in smaller mites. Ebermann [37] attributes this to the fact that the larger mite can swallow the egg intact and, as a result, the oncosphere is unable to hatch before the egg passes through the alimentary tract of the mite. Known intermediate hosts for tapeworms are species of the families Galumnidae, Haplozetidae, Oribatulidae, Scheloribatidae [38], Ceratozetidae, and Oppiidae [29]. A completed list of oribatid species implicated as putative intermediate hosts is provided in [29,31]. Based on our results, we can extend this list to include a new intermediate host species, *Tectocepheus velatus sarekensis*.

Rather surprising were the results of molecular analyses: in two pooled samples of *Tectocepheus velatus sarekensis* from the TD study site, the DNA of *Andrya cuniculi* and unidentified closely related tapeworm species was documented. Based on the results of the coprological analysis demonstrating the presence of *Moniezia* spp. eggs in 13.1–43.3% chamois faces and *Ctenotaenia marmotae* eggs even in 71.1% of marmot faeces, we expected to detect primarily these two species. Importantly, four specimens of the species *Ceratozetes gracilis*, *Edwardzetes edwardsi*, *Scheloribates laevigatus*, and *Trichoribates novus*, in whose abdominal cavity we morphologically detected the presence of cysticercoid, were not suitable for molecular analyses due to DNA destruction after exposure to lactic acid. Therefore, it was not possible to determine the larval stage of the tapeworms they contained. However, during the autopsy of chamois performed during the study period, two adult tapeworms were obtained and identified molecularly as *Moniezia benedeni* (authors, unpublished data). Therefore, we can assume that this species is present in the Tatra chamois. This is also confirmed by earlier findings from the same area [1,39]. In Tatra marmots in Slovakia, *Ctenotaenia marmotae* and *Paranoplocephala transversaria* have been previously documented [40]. An anoplocephalid cestode *C. marmotae* is the most prevalent and abundant helminth of the Alpine marmot throughout its whole range [41]. In the Austrian population of the species, 90.1% of examined individuals were infected with the tapeworm [42], and in 38–100% marmots from 10 study areas in Austria and Switzerland [43], in consistence with our findings of 71.1% of marmot faeces containing *C. marmotae* eggs. Although a variety of endoparasites have been described for the alpine marmots in the Alps, e.g., [44,45,46], in the alpine marmots in the Pyrenees the endoparasitic fauna was not as diverse [47], which is comparable to our findings and findings of other authors from the same area [6]. It is important to note that, since the 18S rRNA gene sequences for this species are not available in the GenBank, the unidentified species related to *Andrya cuniculi* in sample 178 may represent either this species or *A. rhopalocephala*. As judged by recently published molecular phylogenetic trees based on cytochrome oxidase I subunit and NADH dehydrogenase I subunit genes, *Ctenotaenia marmotae* is closely related to *Andrya* [48].

The recent study on gastrointestinal parasites of the *Tatra chamois* revealed a significant difference in *Moniezia* spp. occurrence in different regions of the Tatra Mts. The positivity was significantly higher in samples from Western Tatra and High Tatra Mts. (17.2% and 25.4%, respectively) than in Belianske Tatra and the Polish part of the Tatra Mts. (5.0% and 6.1%, respectively) [6]. Within our research, which was carried out five years later, we observed a different situation, since the occurrence of *Moniezia* spp. eggs was statistically significantly higher in the Lomnické sedlo study site (High Tatra) than in Velická dolina (High Tatra) and Tomanovská dolina (Western Tatra), but contrary to the previous study, the tapeworm eggs were also significantly more frequent in the Dolina Waksmundzka in the Polish part of the Tatra Mts. While in the previous study chamois faecal samples were collected from several localities on the Polish side of the Tatra Mountains and not exclusively from this locality, we assume that the introduction of the parasite occurred through the migration of chamois between localities, mainly on the Polish side of the Tatra Mts. This may be connected with extraordinary numbers of chamois in recent years. Although each herd generally moves within its territory according to certain regularities and changes its habitat little as a part of the daily migration, during the seasonal migration it moves over a wider area, which generally does not extend beyond the territory of one or two adjacent valleys. Extraordinary circumstances cause irregular migration, including changes in the positions of individual chamois or entire herds when pursued by enemies or as a result of the concentration of tourism in mountainous areas [49].

The migration of animals, in this instance the European hare, to higher mountain positions may have been the cause of our unexpected finding of *Andrya cuniculi* in the Tomanovská dolina locality. From the Tatra Mts. territory, the species *Andrya rhopalocephala* (Riehm 1881) in *Lepus eauropaeus* was recorded in the past [3]. *A. cuniculi* has been described in the wild rabbit *Oryctolagus cuniculus* [50], which does not occur in the Tatra Mts., and [51] confirmed this tapeworm only in higher elevations (the authors did not specify the elevation), which could consolidate with our finding from higher altitude at the site Tomanovská dolina (1795–1951 m asl). The European hare is a typical steppe species that prefers open habitats, often staying at the edges of forests. It penetrates valleys up to the upper forest boundary. According to [52], the upper limit of its hypsometric distribution in the High Tatra Mts. runs through the subalpine vegetation stage at about 1600 m asl; [53] gives the upper limit of occurrence in the Western Tatra up to 1200 m asl, in the High Tatra 1300–1500 m asl. In the Polish part of the Tatra Mts., it was observed on the summit of Giewont (1849 m), Kończysta (2193 m), and Woloszyna (2155 m) [54,55]. As there is an alpine meadow with rich vegetation in the Tomanovská dolina study site, where infected oribatids originated from, the most plausible explanation is that, especially in the summer months, the hare also enters the area, contaminating the environment with *A. cuniculi* eggs. The life cycle of *A. cuniculi* is currently not yet fully known [51]. On account of the relatedness of the species to *Paranoplocephala* and *Anoplocephaloides*, confirmed by the morphological similarity in the study of Haukisalmi and Wickström [56], and also by our sequence analyses, we can report that the confirmed intermediate host of the tapeworm in the Tatra Mountains is *Tectocepheus velatus sarekensis*.

It has been proved that critical environmental factors affecting oribatid mites distribution are: temperature, soil moisture, solar radiation, food availability, and soil depth [29]. The analysis conducted in this study has revealed significant differences in the species composition of mites and their abundance between the two studied localities, Lomnické sedlo and Velická dolina. Despite no significant differences in the soil temperature and humidity of the soil between the two locations, the mite communities in each place differed significantly, allowing the hypothesis that these factors may not be the primary drivers of mite distribution in the areas. However, it is essential to note that the factor of locality, which was included in the PERMANOVA model, explains only 26% of the variation observed. This suggests that additional, unmeasured variables may influence the pattern of the oribatid mites communities. So, it is essential to consider the limitations of this study when interpreting the results. Further research is needed to identify and measure the influence of other potentially significant factors on communities of Oribatida in these areas.

Though in our research we were not able to confirm molecularly the occurrence of *Moniezia* spp. and *Ctenotaenia marmotae* larval stages, we identified morphologically five intermediate host species of oribatid mites in the Tatra Mountains area. Our research confirmed the competence of *Tectocepheus velatus sarekensis* as an intermediate host of the tapeworm *Andrya cuniculi*. This is the first record of this oribatid mite species as an intermediate host of anoplocephalid tapeworm, as well as the first report of *Andrya cuniculi* occurrence in the territory of the Tatra Mountains, confirmed by molecular methods.

## Figures and Tables

**Table 1:** Basic characteristics of the studied sites in the Tatra Mts., the positivity of faecal samples for tapeworm eggs, species diversity, and abundance of oribatid mites (LS—Lomnické sedlo, VD—Velická dolina, TD—Tomanovská dolina, DW—Dolina Waksmundzka).

Localities	LS	VD	TD	DW
GPS coordinates	49°11′369″ N 020°12′974″ E	49°09′959″ N 020°09′041″ E	49°13′420″ N 019°54′299″ E	49°23′291″ N 20°04′836″ E
Altitude (m)	1886–2293	1712–1961	1795–1951	1770–2186
Average soil temperature	10.5 °C	9 °C	11.3 °C	NA
Average soil humidity	18.4%	30.3%	22%	NA
Geomorphological subdivision of Mts.	High Tatra	High Tatra	Western Tatra	Polish Tatra
Cardinal direction	southeast	south-southwest	southwest	east-northeast
Bedrock	granite	granite	limestone	granite
Occurrence of the species studied	chamois	chamois/marmot	chamois	chamois
*Moniezia*/*Ctenotaenia* positivity (%)	33.6/NA	16.2/71.1	13.1/NA	43.3/NA
95% CI	25.5–43.1	10.1–24.2/54.1–84.6	0.1–16.2	25.5–62.6
Oribatid species diversity (No. of taxa)	72	74	68	50
Abundance (ex.)	7011	6497	2930	2799

**Table 2:** The list of oribatid species recorded as intermediate hosts, based on literature sources [28,29,30,31] collected from four study sites in the Tatra Mts., with the recorded number of individuals at each site (* indicates a finding of cysticercoid in our study, underlined is a new confirmed intermediate host species).

Species	LS	VD	TD	DW
*Adoristes ovatus* (C.L. Koch 1839)	1	10	-	6
*Achipteria coleoptrata* (Linnaeus 1758)	5	40	7	100
*Cepheus cepheiformis* (Nicolet 1855)	9	-	-	-
*Ceratoppia bipilis* (Hermann 1804)	16	25	8	36
*Ceratozetes gracilis* (Michael 1884)	-	11	2	1 *
*Edwardzetes edwardsi* (Nicolet 1855)	1	10 *	-	-
*Hermannia gibba* (C.L. Koch 1839)	372	825	16	65
*Liacarus coracinus* (C.L. Koch 1841)	7	23	-	-
*Liebstadia similis* (Michael 1888)	68	45	39	-
*Oppiella nova* (Oudemans 1902)	2	16	25	-
*Pilogalumna tenuiclava* (Berlese 1908)	100	28	-	278
*Scheloribates laevigatus* (C.L. Koch 1835)	356	536 *	34	133
*Scheloribates latipes* (C.L. Koch 1844)	34	25	-	11
*Scheloribates pallidulus* (C.L. Koch 1841)	-	-	-	5
*Tectocepheus* *velatus sarekensis* (Trägårdh 1910)	484	519	384 *	161
*Trichoribates novus* (Sellnick 1928)	-	-	6 *	9
*Trichoribates trimaculatus* (C.L. Koch 1835)	-	1	1	-

## Data Availability

The data used to support the findings of this study are available from the corresponding author upon request.
